# Notice from Secretary T. S. M. A. to Members

**Published:** 1886-11

**Authors:** F. E. Daniel

**Affiliations:** Secretary; Office Secretary of State Medical Association, Austin, Texas


OFFICE SECRETARY OF STATE MEDICAL ASSOCIATION,
AUSTIN, Texas, November 20, 1886.

SECRETARIES of subordinate and of auxiliary medical associations in Texas, County and District, are urgently requested to furnish this office at as early date as practicable a report of the number of active members of their respective organizations, together with name and address of President and Secretary; also, to report promptly any changes in these offices. This is necessary in order to enable the Secretary to promptly disseminate information of importance emanating from the National, or State Medical Association, as well as to perfect the roll of the latter, and to regulate representation, etc.

F. E. DANIEL, Secretary.

---

MEMBERS OF THE TEXAS STATE MEDICAL ASSOCIATION, who find errors or omissions in their record in the roll of members in the transactions just issued, will do themselves justice and the secretary a favor, if they will promptly point out such, and thus enable the publishing committee, another year, to correct it. Many have never furnished the necessary data for their record, as will be seen by the roll. If any members have not received the transactions, they will report promptly to this office.

